# Microfluidic Flow Injection Immunoassay System for Algal Toxins Determination: A Case of Study

**DOI:** 10.3389/fchem.2021.626630

**Published:** 2021-03-04

**Authors:** Lorenzo Celio, Matteo Ottaviani, Rocco Cancelliere, Alessio Di Tinno, Peter Panjan, Adama Marie Sesay, Laura Micheli

**Affiliations:** ^1^ Departement of Chemical Sciences and Technologies, University of Rome Tor Vergata, Rome, Italy; ^2^ Department of Analytical Chemistry, University of Turin, Turin, Italy; ^3^ Department of Electrical and Information Engineering, University of Cassino and Southern Lazio, Cassino (FR), Italy; ^4^ Measurement Technology Research Unit, Oulu University, Kajaani, Finland; ^5^ Essi Tech d.o.o., Ljubljana, Slovenia; ^6^ Wyss Institute of Bioinspired Engineering, Harvard University, Boston, MA, United States

**Keywords:** FI-IA, algal toxins, saxitoxin, immunoanalytical system, microchip flow-chamber system

## Abstract

A novel flow injection microfluidic immunoassay system for continuous monitoring of saxitoxin, a lethal biotoxin, in seawater samples is presented in this article. The system consists of a preimmobilized G protein immunoaffinity column connected in line with a lab-on-chip setup. The detection of saxitoxin in seawater was carried out in two steps: an offline incubation step (competition reaction) performed between the analyte of interest (saxitoxin or Ag, as standard or seawater sample) and a tracer (an enzyme-conjugated antigen or Ag*) toward a specific polyclonal antibody. Then, the mixture was injected through a “loop” of a few μL using a six-way injection valve into a bioreactor, in line with the valve. The bioreactor consisted of a small glass column, manually filled with resin upon which G protein has been immobilized. When the mixture flowed through the bioreactor, all the antibody-antigen complex, formed during the competition step, is retained by the G protein. The tracer molecules that do not interact with the capture antibody and protein G are eluted out of the column, collected, and mixed with an enzymatic substrate directly within the microfluidic chip, via the use of two peristaltic pumps. When Ag* was present, a color change (absorbance variation, ΔAbs) of the solution is detected at a fixed wavelength (655 nm) by an optical chip docking system and registered by a computer. The amount of saxitoxin, present in the sample (or standard), that generates the variation of the intensity of the color, will be directly proportional to the concentration of the analyte in the analyzed solution. Indeed, the absorbance response increased proportionally to the enzymatic product and to the concentration of saxitoxin in the range of 3.5 × 10^–7^–2 × 10^–5^ ng ml^−1^ with a detection limit of 1 × 10^–7^ ng ml^−1^ (RSD% 15, S N^−1^ equal to 3). The immunoanalytical system has been characterized, optimized, and tested with seawater samples. This analytical approach, combined with the transportable and small-sized instrumentation, allows for easy *in situ* monitoring of marine water contaminations.

## Introduction

**FIGURE 1:**
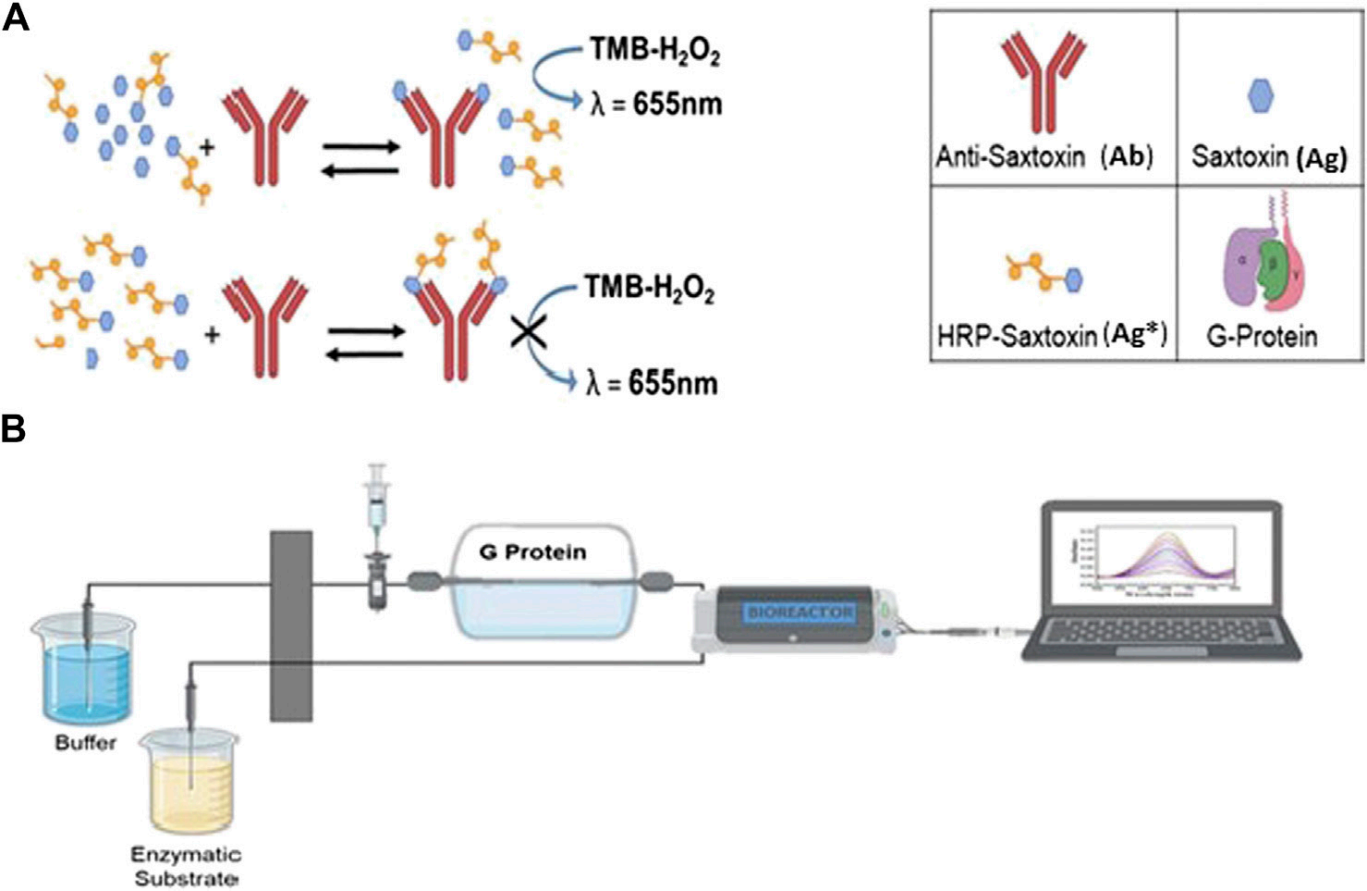
Scheme of FI-IA approach where the bioreactor is in line with the lab-on-chip cartridge and connected to a laptop. In particluar, 1b: **(A)** offline incubation between STX, HRP-STX, and PAb; **(B)** injection of the mixture in the FI-IA system.

The development of rapid and sensitive analytical methods for the determination of trace analytes in liquid samples is an important goal to be achieved in analytical chemistry. In the last decade, the demand for continuous monitoring systems has increased in clinical, pharmaceutical, and environmental chemistry, thus favoring an ever-greater development of high throughput in flow systems (Fintschenko and Wilson, 1998; Ishimatsu et al., 2020). Flow analysis methods have been widespread in the field of chemical analysis since the middle of the last century when Růžička and Hansen first introduced the term “Flow Injection Analysis (FIA)” (1975) (Růžička and Hansen, 1975). This is considered the cornerstone of a new paradigm in analytical chemistry (McKelvie, 2008). These publications gave rise to FIA and with it to an entire field of research that, over the following 3 decades, has involved thousands of researchers, which to date has resulted in more than 16,000 publications in the scientific literature (McKelvie, 2008; Rocha and Zagatto, 2020). This technique is generally based on straightforward and cost-effective manifolds with the possibility of being adapted to distinct analytical requests (Passos et al., 2015). Flow injection immunoassays (FI-IA), combining FIA technique with immunoassay (IA), provide specific detection and allow rapid and reliable determination. Immunological methods, which are based on the specific interaction between the antibody and antigen, exhibit some significant advantages over classical flow injection techniques such as simple layout, inexpensiveness, and, most importantly, sensitivity and specificity. The merits of the two methods are exploited simultaneously. The aforementioned system fits well with applications in the determination of different analytes in real samples. Pesticides (Krämer and Schmid, 1991), food (Waseem et al., 2013), tumor markers determination (Wu et al., 2006; Zhang et al., 2013), control of microbial growth (Wang et al., 2012), monitoring of chemicals in wastewater (Shi et al., 2018), and evaluation of harmful algal blooms in seawater samples (Shitanda et al., 2009) are among the most important and worthy of these applications. Algal toxins are harmful organic molecules released by naturally decaying or degrading unicellular algae (algal toxins are primarily produced in detrimental concentrations during harmful algal bloom) (Vilariño et al., 2013). This overcrowding can give rise to a phenomenon of accumulation in seawater animals (mollusks, shellfish, and fishes), causing intoxication along the entire food chain that involves cetaceans, birds, other mammals, and finally humans. Over the past few years, many different analytical approaches have been explored in the area of algal toxin detection from environmental samples, including liquid chromatography coupled with mass-spectroscopy (LC-MS) (Vilariño et al., 2013), high-performance liquid chromatography (HPLC) (Van Egmond et al., 1994), enzyme-linked immunosorbent assay (ELISA) (Micheli et al., 2002; Yu et al., 2002; Petropoulos et al., 2019), and electrochemical biosensors (Zhang et al., 2018). Most of the research has been focused on the saxitoxin (STX) and STX-related compounds, as they are found to be more common in neurotoxic paralytic shellfish toxins (PSTs) (Deeds et al., 2008). These PSTs represent a group of naturally occurring neurotoxic alkaloids. STX is the most researched PST to date, and since its discovery in 1957, 57 analogues have been described. Intoxication with PSTs may result in a serious and occasionally fatal illness known as paralytic shellfish poisoning (PSP) (Anderson et al., 1996; Falconer, 1993; García et al., 2004): this illness manifests itself when PSTs reversibly bind voltage-gated Na^+^ channels in an equimolar ratio. This is mediated by the interaction among different functional groups: the positively charged guanidinium groups of STX interact with negatively charged carboxyl groups of the Na^+^ channel, thereby blocking the pore. The threat of PSP is not only a major cause of concern for public health but also deleterious to the economy. Outbreaks of PSTs often result in the death of marine life and livestock and the closure of contaminated fisheries. Moreover, the frequent disbursement required for running monitoring programs, together with the aforementioned critical issues, presents a major economic burden around the world (Guy and Griffin, 2009; Ferrão-Filho Ada and Kozlowsky-Suzuki, 2011). The officially prescribed and validated methods for STX detection in the European Union were the mouse bioassay (MBA), based on the injection of 1 ml test solution in live mouse and observing the time from injection to death (with a limit of detection ca. 40 mg STX eq/100 g shellfish) (Cusick and Sayler, 2013), and the Association of Official Analytical Chemists (AOAC) official method 2005.06 (Lawrence method) with a precolumn derivatization and fluorescence detection (LC-FLD) (He et al., 2005). The liquid chromatography postcolumn oxidation (PCOX) method was acknowledged by AOAC in 2011 as the official method. The Interstate Shellfish Sanitation Conference (ISSC, United States) certified two commercially available immunological methods-based products, namely, Abraxis ELISAs and Scotia LFAs as “Approved Limited Use Method for Marine Biotoxin” (Li and Persson, 2021). For drinking water, the American EPA listed Enzyme-Linked Immunosorbent Assays (ELISA) together with LC-MS for PST detection [United States Environmental Protection Agency (EPA), 2019]. In parallel, four immunological categories (immunoassay, immunosensor, lateral flow immunoassay, and radioimmunoassay) were developed for the detection of algal toxins in shellfish and water. These methods for PSTs are suitable for field testing because the extraction and detection are simple. They are also quick to perform and of relatively low cost per sample, need minimal equipment, and are relatively simple and sensitive. They are used widely in the food industry in a variety of countries, with rapid results enabling real-time decisions on the fate of harvested shellfish products (Anfossi et al., 2018; European Food Safety Authority (EFSA), 2009; Micheli et al., 2002). Recently, several interesting overviews about analytical methods developed for the detection of algal toxins are published with a comparison of the advantages and the limits. The conclusions of these overviews are important for developing fast screening methods that should be combined with highly sensitive and accurate analytical methods such as liquid chromatography/liquid chromatograph-mass spectrometry (LC/LC-MS) for confirming the results. LC/LC-MS is able to separate and detect all major PSTs in ranges between 14.95 and 299 and 1.5–8.97 ng L^−1^ (Dell’Aversano et al., 2005; Botana et al., 2013). Further developments of this technique have enabled detection limits in concentration ranges of 1.5–14.95 and 7.48–59.80 ng L^−1^, with excellent linearity; however, this technique was not applicable out of the laboratory (Halme et al., 2012; Cusick and Sayler, 2013). Tian et al. (2020) reported an overall vision about the recent progress of optical and electrochemical biosensors developed for the detection of shellfish toxins in food and drinking water, showing the advantages of the strategy, analyte, sensing unit, method, and property for their future application in field fast screening (Tian et al., 2020). For a prompt response to potential pollution of seawater, limited poor information is present about continuous monitoring of the health of seawater in terms of the presence of PSTs, in particular STX (Pöhlmann and Elßner, 2020). Innovative methods in aquatic toxins detection are mainly focused on the production of rapid, easy-to-use, and highly sensitive multianalyte detection for on-site detection of the dangerous agent (Campbell et al., 2011; Stroka, 2011). An easy-to-perform, high-throughput assay would be a highly valuable tool considering the increase in the number of samples to be processed by routine testing laboratories. In this article, we aim at demonstrating the applicability of the FI-IA technique connected to a microfluidic system to the determination of STX in seawater samples. This kind of microfluidic systems avoids using high volumes of samples and reagents. This allows decreasing the cost of chemical analysis and reducing wastes. Moreover, injecting the analyte directly in an enclosed environment averts interaction between analyte and external interferents, giving more reliable results and decreasing analytes loss. Lastly, the sample travels throughout the system in a short time range, thus giving high performance and reproducibility. The proposed FI-IA system consists of a bioreactor in line with a lab-on-chip (LOC) microfluidic system for reagents mixing and colorimetric detection (Figure 1 and Supplementary Figure S1).

This monitoring tool allows a detection limit (LOD) for STX as low as 1 × 10^–4^ ng L^−1^, with a working range equal to 3.5 × 10^–4^–2 × 10^–2^ ng L^−1^ in seawater samples with a column shelf life of 38 sequential measurements directly *in situ*. The main feature of this system, as well as its greatest advantage, is the application of a small volume sample (25 µL loop), allowing for small consumption of reagents and reducing waste. The preliminary study, presented here, illustrates its potential of being used as a field-deployable portable analytical device able to work *in situ* and with continuous water samples without any treatment of the sample compared with the chromatographic and immunological methods reported previously. The high sensitivity, low detection limit, and short time of analysis make the proposed system suitable for field assays in which the speed of analysis is one of the most important parameters.

## Materials and Methods

### Chemicals and Bioreagents

Saxitoxin (STX) 100 g L^−1^, horseradish peroxidase (HRP) 150 U mg^−1^, 3,3′,5,5′-tetramethylbenzidine (TMB, enzymatic substrate ready to use), and G protein immobilized on 4% agarose were purchased from Sigma-Aldrich (United States). Polyclonal antibodies anti-STX (PAb) AS111663 was purchased from Agrisera Antibodies (LiStarFish, IT). Sodium azide (NaN_3_), sodium phosphate (NaH_2_PO_4_), sodium carbonate (Na_2_CO_3_), sodium acetate (C_2_H_3_NaO_2_), and Patent Blue V (E131) were purchased from Sigma-Aldrich, United States. Buffer solutions used are 20 mM phosphate buffer solution, pH 7.4; 200 mM sodium carbonate buffer solution, pH 9.6; 1 mM acetate buffer solution, pH 4.4. All reagents are of analytical grade unless otherwise stated.

### Lab-on-Chip Components

The microfluidic chip (PMMA) was fabricated at the Measurement Technology Unit, CEMIS-Oulu (University of Oulu, Finland) using the Arduino Nano Board (Arduino, IT) and spectrophotometric detector compatible with Arduino Board (Arduino, IT). The PMMA microfluidic chip was fabricated using a CO_2_ laser to carve PMMA (black and transparent) and bonded together by double-sided medical grade adhesive tape, 100 um thick. The channels were cut out of the adhesive tape and therefore had a defined depth; that is, they were 100 um deep. Herringbone structure was carved for an additional 100 um into the PMMA, yielding a maximum depth of 200 um.

### Flow Injection Immunoassays System’s Components

The FI-IA system (Supplementary Figure S1) consists of a 25 µL loop (made in-house) and Rheodyne® Model 7125 syringe loading injector (United States), Glass Omnifit® column (length: 2.5 cm; seating capacity: 0.35 ml), and PTFE Frits (pores’ diameter: 25 µm) purchased from Omnifit® (Rockville Center, NY, United States). Gilson Minipuls® 3 pumps (model M312, Gilson, United States), four-way peristaltic pumps and their tubes (PVC pipes compatible with Gilson Minipuls®3), PVC pipes compatible with Gilson Minipuls® 3 (model: F117934, ø = 0.51 mm), PVC pipes compatible with Gilson Minipuls® 3 (model: F117936, ø = 0.76 mm), and PVC pipes compatible with Gilson Minipuls®3 (model: F117938, ø = 1.02 mm) were purchased from Gilson® (France). The column is packed with G protein immobilized on agarose beads (Sigma-Aldrich, United States).

### Principle of Methods

The proposed FI-IA method (Figure 1) for STX quantification consists of an offline incubation of the sample containing STX (Ag) with fixed amounts of anti-STX antibody (Ab) and STX labeled with peroxidase (Ag*); in this mixture, a competition between Ag and Ag* for the binding sites of Ab occurs. After this step, the mixture is injected into a flow system where the separation of free Ag* and the antibody-bound tracer (Ab-Ag*) is performed in a column with the coated G protein. The competition mixture is injected through a six-way injection valve, the same type as those used in chromatography, equipped with a fixed volume loop (25 µL). The column is placed in line with the injection system. The microfluidic system is equipped with two inlet channels, the first one for the solution leaving the bioreactor and the second one for the enzymatic substrate, and a single outlet channel for the enzymatic product. In the proposed system, two microfluidic mixers are present where the solution, eluted by the bioreactor and containing the Ag*, mixes with the enzymatic substrate TMB. After the mixing zone, there is a serpentine incubation channel where TMB is oxidized by the enzymatic reaction. The formed enzymatic product is continuously read through the microchip optically clear channel, by a fixed wavelength filter (655 nm), present in the housing of the microfluidic system, to which a laptop is connected for online absorbance acquisition.

### Proposed System

**FIGURE 2:**
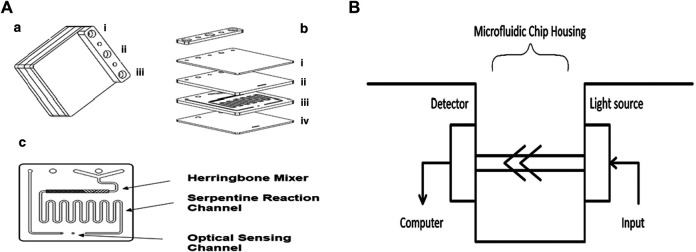
Schematization of the microfluidic lab-on-chip system: **(A)** microfluidic bioreactor and components; **(B)** microfluidic chip detector housing (LED 655 nm and photodiode). In particular, in **(A)**: (a) i) outlet; ii) substrate inlet; iii) sample inlet; (b) lab-on-chip components: i) top plate, ii) black PMMA herringbone etched plate, iii) microfluidic channels, and iv) clear bottom plate; (c) microfluidic mixing chamber, reaction channel, and optical paths.

The complete microfluidic system consists of two peristaltic pumps, a 25 µL loop circuit, which includes a six-way injection valve, a bioreactor, an Arduino Nano Board, and a spectrophotometric detector compatible with the latter, and a microfluidic chip. Each inlet and outlet is linked using PVC tubes and the detector system is connected to a notebook. The microfluidic chip (Figure 2A) consists of four polymethacrylate (PMMA) surfaces (Figures 2A,a,b): the external plates are transparent and 0.25 cm thick, whereas the internal ones are black, opaque, and 0.5 cm thick (1.5 cm full-thickness). The microfluidic path (Figures 2A,c) is engraved by laser: it consists of a Y channel that connects the two inlets (one for the TMB-enzymatic substrate and the other for the analytes eluted from the bioreactor) and mixes the two entering flows by diffusion; a herringbone mixer that mixes the running solution further by mechanically imposing a “turbulent” flow regime; a coil path (which restores the laminar flow regime); the 1 cm long optical detection chamber (Figures 2A,c). The latter is then linked directly to the outlet. The microfluidic path has a depth of 100 µm, except for the herringbone mixer that has 100 µm depth and alternate 200 µm drops. Chip, detector, and valve are located in a 3D printed Acrylonitrile Butadiene Styrene (ABS) housing (Figure 2B).

### Procedures for the Preparation for the Conjugation of STX With HRP

STX was conjugated to horseradish peroxidase (HRP) via the periodate reaction (Micheli et al., 2002). The STX-HRP concentration was evaluated using the Pierce Bicinchoninic Acid Assay, where bovine serum albumin (BSA) was used as a reference protein. The assay was performed on flat-bottom 96-well plate MaxiSorp (NUNC, Roskilde, Denmark) by adding 5 µL of BSA dilutions to 150 µL of BCA reagents to the well plate; following this step, 5 µL of sample was added to 150 µL BCA reagents, and then the whole plate was incubated for 30 min at 37°C and read spectrophotometrically at 570 nm in triplicate. The assay showed a conjugated STX-HRP concentration of 80 µg ml^−1^ of protein. The residual activity was defined by following the ABTS [2,2′-azino-bis(3-ethylbenzothiazoline-6-sulphonic acid) Sigma-Aldrich, United States] protocol adding 1 mM H_2_O_2_ and 14.6 mM ABTS on phosphate buffer 50 mM; the subsequent spectrophotometric measure at 403 nm showed a residual activity of 1.73 U mL^−1^ against 5.02 U mL^−1^ of native protein.

### Bioreactor Column

The bioreactor consists of a borosilicate glass chromatography column (25 mm length, 0.35 ml bed volume) filled with G protein (immobilized onto 4% agarose bead, with a dynamic binding capacity of 20 mg human IgG mL^−1^), housing made.

#### Preparation of the Bioreactor and Its Storage

##### Column Packing

Omnifit® chromatographic column (see *FI-IA System’s Components*), applied in the FI-IA system, is packed by means of 40 mg of agarose particles activated by G protein and solubilized in 20 mM phosphate buffer, pH 7.4. The buffer solution is kept flowing for 1 h to make sure the column packing is as homogeneous as possible and stored at 4°C after the addition of 30 mM NaN_3_ prepared in phosphate buffer. Before using the bioreactor, it was washed with phosphate buffer for 15 min in order to remove all NaN_3_ present.

##### Column Preservation

When the analysis was over, packed chromatographic column is cleaned up with a solution of 30 mM of NaN_3_ in 10 ml of 20 mM phosphate buffer, pH 7.4, and then preserved at 4°C. This is done in order to avoid mold development or bacterial attacks.

##### Preparation of the FI-IA Microfluidic Tool

The system is assembled by connecting with its components (two peristaltic pumps, the “housing” part containing the detector and the injection valve, the bioreactor, and the microfluidic system). After the full system is assembled, the carrier buffer solution is left to flow for 60 min before starting the measurements. This is necessary to fill the whole system and eliminate any air bubbles trapped inside.

##### Sample Injection

A 200 µL of the solution, containing the sample and the immunoreagent (Ab and Ag*), is injected into the system using a microsyringe; in this way, the loop (25 µL) is being loaded with the sample solution. Then, 20 mM phosphate buffer solution, pH 7.4, as a carrier, is fluxed at 0.1 ml min^−1^, using the peristaltic pump, dragging the mixture through the column. As soon as the whole sample is injected, the loop is opened and kept that way for 4 min, thus beginning the analysis. The peristaltic pump, set to maximum flow speed, carries the immunocomplex and the other bioreagents through the bioreactor. Thus, all the volume flows through the column and gets to the microfluidic system, in which it gets mixed with the enzymatic substrate (TMB) and gets detected with the detector set at 655 nm. After 20 min from the previous injection and only when the system is cleaned (done directly while performing the analysis, after 10 min from the injection), it is then possible to proceed with the next injection.

### FI-IA Procedure for Saxitoxin Determination

FI-IA procedure consists of two fundamental steps: offline incubation of the sample (or the standard) for the competitive assay and its injection in the “flow system.” The first step consists in the addition of solution containing the sample into a vial (or the standard, Ag) with a fixed amount (1:75,000 v v^−1^) of primary anti-STX polyclonal antibody (PAb) and STX-HRP (Ag*) (1:600 v v^−1^). The solution is then incubated for 2 h. In this condition, a competition reaction is carried out between Ag and Ag* toward the binding sites of the specific antibody (Ab). After the incubation step time, this mixture is injected through the flow system for the analysis (second step). Once the mixture passes through the bioreactor, all the antibodies are withheld by G protein and bioreagents that have not been caught by the antibody (large excess Ag*) are eluted from the column. These molecules proceed until they enter one of the two inlets of the microfluidic system, whereas in the other inlet, enzymatic substrate is flowing through. Later, both the solutions enter in the first microfluidic mixer, the T sensor, where they undergo a first mixing by diffusion. If the conjugated Ag* is present, a colorimetric variation, due to the development of enzymatic product (oxidized TMB, blue), will happen. Conversely, if the desired analyte (Ag) is absent within the sample, all the conjugated Ag* will remain bound to the antibody and will be retained inside the column, giving no colorimetric change. By increasing Ag concentration, competition will occur between Ag and Ag* for the binding of the antibody sites. The free Ag*, not retained in the bioreactor, will be eluted and will react with the enzymatic substrate (TMB), giving a colored enzymatic product proportional to the concentration of the sample (or the standard). After the first mixer, which links both the inlets into one microfluidic channel, a second mixer is present, the herringbone mixer, which further mechanically mixes analytes and reagents by changing flow’s nature from laminar to turbulent. After this last mixing procedure, a long microfluidic channel allows the flow to laminar flow once again, giving a further and definitive homogenization of the enzymatic product. Eventually, the fluids get to the detector chamber, in which absorbance is measured periodically at 1 s intervals.

### Analytical Parameters Calculation

Measuring occurs by connecting the system to a notebook in line with the LOC. The notebook itself powers the Arduino Nano Board and the linked optical detector. Through a firmware implemented on the same board, the light source emits a light ray at fixed wavelength (655 nm) every second. With the same time range, the detector (placed in front of the source and on the same axis of the light ray) measures the intensity of the radiation. The enzymatic reaction which occurs between the enzymatic substrate (TMB) and the labeled antigen (Ag*), eluted from the column, produces a signal output. The intensity of the latter is directly proportional to the concentration of the eluted Ag* and directly proportional to the concentration of injected analyte sample. The unknown analyte concentration is determined by using a calibration curve previously built, analyzing standard samples of known concentration of the analyte itself.

#### FIA Peaks Analysis

Once collected from continuous analysis, data are plotted to give a peak-like shape of which height (H), width (W), and area (A) contain the analytical information of interest. Moreover, the detector gives a linear and instantaneous answer because once the microfluidic channel has been fully covered, analytes are homogeneously mixed thanks to diffusion and turbulent flow (which is then restored to laminar before entering the detector). Thus, there is no limitation in choosing one of the three parameters. In our experiment, height (H) at peak maximum is the desired parameter, and it coincides with absorbance maximum because it is easily measurable and strictly related to the detector’s reading, particularly with absorbance measuring. The response of this latter, achieved by detecting the amount of colored enzymatic product in the detection chamber, has a bell function-like shape. In fact, it is a usual procedure in FIA to treat these data by making a fit with a three-parameter Gaussian distribution (Růžička and Hansen, 1988). Thus, a no-linear fitting is made from collected and plotted data by using the following equation (Eq. 1):

$$y=a\cdot e^{\left[-5\;{\left(\frac{x-x_{0}}{b}\right)}^{2}\right]},$$ (1)

where **a** is the normalizing constant equal to $1/\sigma \cdot \sqrt{2\pi }$; b as doubled variance is equal to $\sqrt{\frac{1}{\text{n}}\sum_{\text{i}=1}^{\text{n}}{\left({\text{x}}_{\text{i}}-\;\bar{\text{x}}\right)}^{2}}$, where x_i_ is the *i*th measure, $\bar{\text{x}}$ is the measures’ average, and **x**_**0**_ is desired value, which corresponds to the x-coordinate of the absorbance maximum of a determined peak. Thus, the desired parameter is x_0,_ which enables us to determine the peak’s height and the absorbance maximum. Moreover, x_0_ is directly proportional to the concentration of the enzyme-antigen complex eluted from the column and proportional to the concentration of the injected analyte. Reproducibility measures have been conducted by subsequent injections of the same analyte. From the collected data, the relative standard deviation (RSD%) has been calculated and expressed in percentage terms.

#### FIA Peaks Analysis Applied to the Determination of STX

Absorbance response depends on concentration and gives a sigmoidal trend. This latter can be defined with a 3-parameter logistic function (Eq. 2), which is characteristic of immune-enzymatic assays.

$$y=\frac{a}{1+{\left(\frac{x}{x_{0}}\right)}^{b}},$$ (2)

with a, b, and x_0_ values are equal to Eq. 1. Moreover, to allow a comparison between different calibration curves, obtained absorbance values have been converted as percentages by applying the following equation (Eq. 3):

$$\%\frac{A}{A_{sat}}=100x\frac{A-A_{0}}{A_{sat}-A_{0}},$$ (3)

where A is equal to the detected absorbance when the analyte is present; A_sat_ and A_0_ are, respectively, absorbance values at the zero competition and at saturating concentration of the analyte.

The limit of detection (LOD) is estimated from the analysis of ten different samples in which the requested analyte is not present (blank); thus, with the obtained current values, the standard deviation (SD) is estimated. Thus, the results found were included in the formula (detection limit: LOD = A_NC_ − 3*σ*) in which SD_nc_ and A_NC_ are the standard deviation and absorbance of the no competition point (no Ag), respectively.

#### Seawater Sampling

The calibration curve was achieved through a matrix matching method with surface seawater sampled from Santa Severa Bay (RM, IT) and Acquafredda bay (PZ, IT). The surface seawaters were sampled with 500 ml PET Water Sampling Bottles (Sterilin, Thermo Fisher Scientific, United States) about 50 m off the coast. The samples were filtered on Millex-GV (hydrophilic PVDF 0.22 µm membrane, Sigma-Aldrich, United States) and stored at 4°C until analysis. Seawater pH and conductivity were measured with a pH meter (Xs Instruments, Modena, IT). Santa Severa seawater showed a 7.85 pH and 57.00 mS conductivity, and Acquafredda seawater a 7.51 pH and 59.10 mS conductivity.

## Results and Discussion

### Study of the of Operational Parameters

The aim of this study is to combine the selectivity and sensibility of the Fl-IA method with the repeatability and the continuous online analysis in microfluidic systems ready to use in the field. Before connecting the bioreactor to the chip, several parameters have been studied. Flow rate is one of the fundamental parameters needed to correctly operate this microfluidic system. Due to the structural features of the chip itself, the flow rate cannot be higher than 0.15 ml min^−1^, while the bioreactor is able to work at higher flow rates. The first set of tests has been carried out to determine the revolutions per minute (RPM) at which each peristaltic pump can operate with the most efficiency (Table 1) with different PVC tubes’ diameters.

**TABLE 1:** Flow rate optimization parameters.

Internal tube diameter, (ø) (mm)	RPM	Average time, <t> (s)	Flow rate, v_*φ*_ (ml min^−1^)	RSD (%)
0.51	1.00	3.33 + 0.09	0.03	3
	1.18	2.50 + 0.06	0.04	2
	3.33	1.02 + 0.02	0.10	2
0.76	1.50	1.067 + 0.006	0.09	0.6
	1.75	0.913 + 0.006	0.11	0.7
	2.38	0.667 + 0.006	0.15	0.9
1.02	1.00	1.133 + 0.006	0.09	0.5
	1.10	1 + 0	0.10	0
	1.20	0.96 + 0.2	0.10	21
	1.40	0.873 + 0.005	0.11	0.6

**FIGURE 3:**
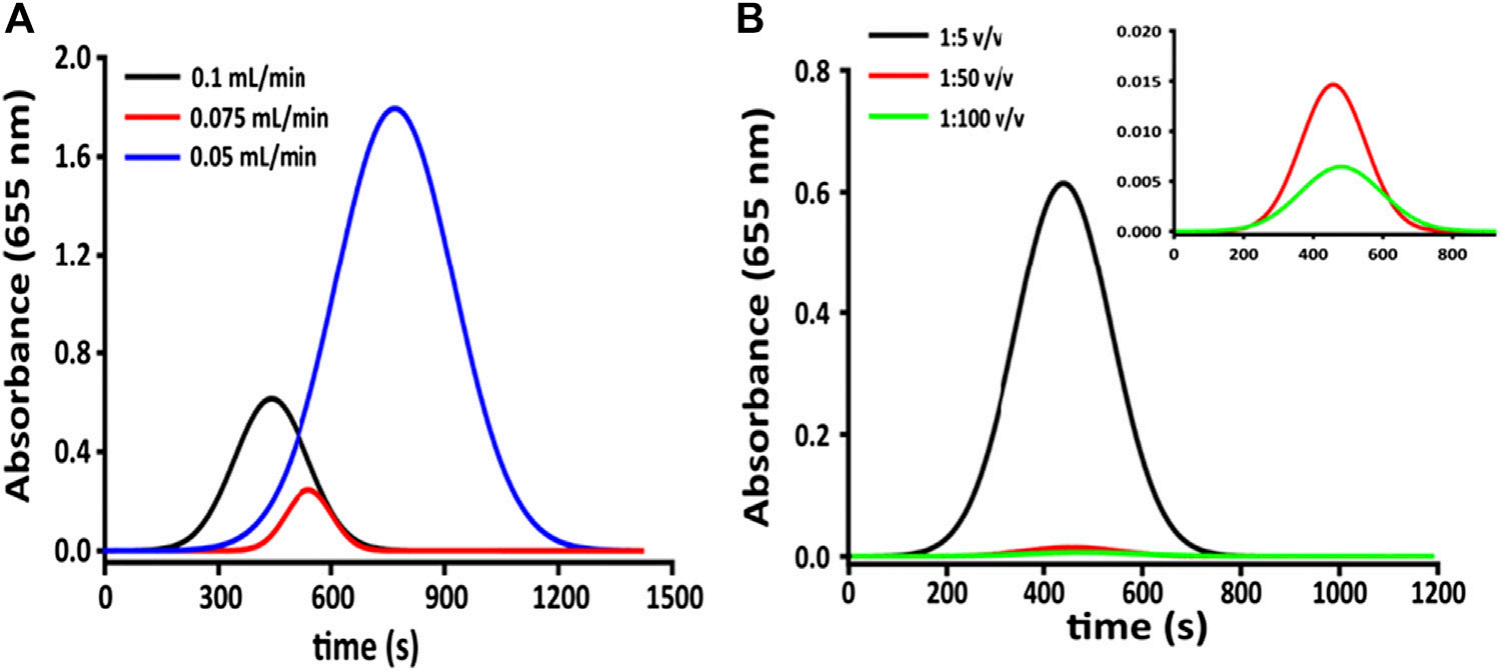
**(A)** Detector response (variation of absorbance) at fixed dilution of the colored substrate and different flow rates; **(B)** detector response (in variation of absorbance) at different dilutions and fixed flow rate (0.1 ml min^−1^).

Tubes of 0.51 mm diameter with an RSD% equal to 2%, were used for the inlet flow, whereas for the outlet flow, larger pipes of 1.02 mm internal diameter were chosen to drop counter pressure interference. The best detector response (in variation of absorbance), reported in Figure 3A, has been obtained by studying three different flow rates according to mechanical limits (0.05, 0.075, and 0.10 ml min^−1,^ respectively). The repeatability of the measurement, calculated on subsequent injections of the same dye dilution at 0.1 ml min^−1^ (good agreement between reproducibility and analysis time) is around 4%. The second set of tests has been conducted to evaluate the response of the microfluidic detector. Multiple injections of a blue food coloring (Patent Blue V E131) at different dilutions (1:100, 1:50, and 1:5 v v^−1^) have been used for the microfluidic detector evaluation since this latter is set at 655 nm. As can be seen in Figure 3B, dilution of 1:100 v v^−1^ gives a signal too weak to be acceptable. That is due to the low concentration of the analyte (the noise given by the moving flow is prevailing over the signal).

### HRP Concentration Optimization

**FIGURE 4:**
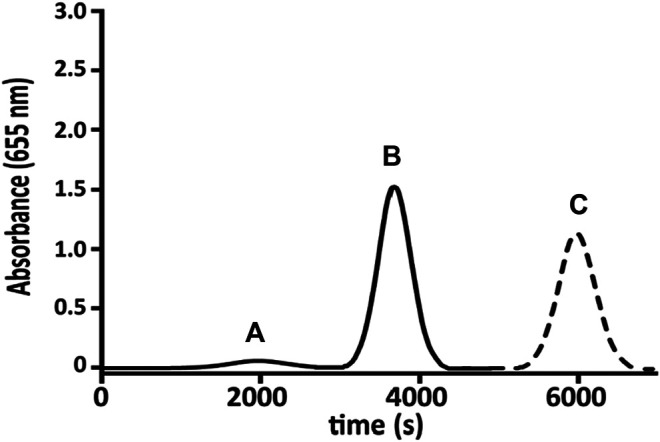
Gaussian regression of several responses [0.0001 **(A)**, 0.001 **(B)**, and 0.1 µg ml^−1^ **(C)**], sequentially injected in the full system at 0.1 ml·min^−1^.

The response of microfluidic system has been evaluated, by a simulation of the immune-enzymatic assay, by measuring of enzymatic substrate flowing in channels I and II of Figure 3B, several concentrations of antigen labeled with HRP (Ag*) and TMB substrate, respectively. As reported in Figure 3B, the enzyme was mixed with its substrate in the herringbone mixer giving the enzymatic product, TMBox, colored in blue and measured in the optical sensing channel. The LOD of TMBox of this system has been calculated measuring multiple sequential injections of different HRP concentrations (0.0001, 0.001, and 0.1 µg ml^−1^), in 20 mM phosphate buffer, pH 7.4, and mixed with a fixed amount of TMB substrate (enzymatic substrate ready to use, Sigma-Aldrich, United States). The results (Figure 4) showed that 1 ng ml^−1^ is the concentration limit for the sensitivity of the detector.

**FIGURE 5:**
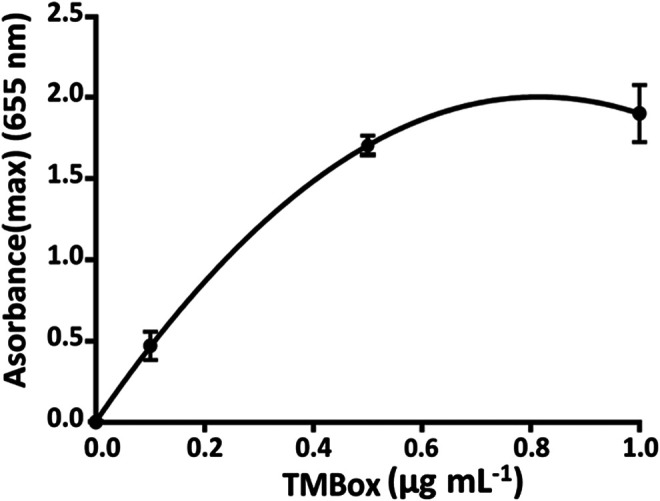
Calibration curve of HRP in the microfluidic system using 0.1 ml min^−1^ flow rate.

Up to 1 µg ml^−1^, the concentration of TMB is unstable (TMS is photosensitive) changing the color from blue to orange. A calibration curve of TMBox (from 0 to 1 µg ml^−1^) was realized using different concentrations of HRP and fixed amount of TMB substrate as such (Table 2). Each measure has been made in triplicate to evaluate the working range and the repeatability without stopping the flow (Figure 5).

**TABLE 2:** Calibration curve parameters for TMBox in the function of the concentration of HRP using microfluidic system.

Concentration (µg ml^−1^)	<Absorbance>	RSD (%)
0	0.05 ± 0.04	2
0.1	0.47 ± 0.08	17
0.5	1.70 ± 0.04	2
1	1.90 ± 0.18	10

## FI-IA Optimization Using Bioreactor

In order to develop the FI-IA system before its connection with the fluidic microchip, several analytical parameters (flow rate, antibody concentration, and interaction time in the bioreactor) were studied, collecting the solution eluted of the bioreactor up to a maximum volume of 200 µL. The solution was collected in tubes and read on the spectrophotometer at 655 nm (only TMBox) and 450 nm (after blocking with 100 µL of H_2_SO_4_).

### Binding Curve and Competition Time Using Bioreactor

**FIGURE 6:**
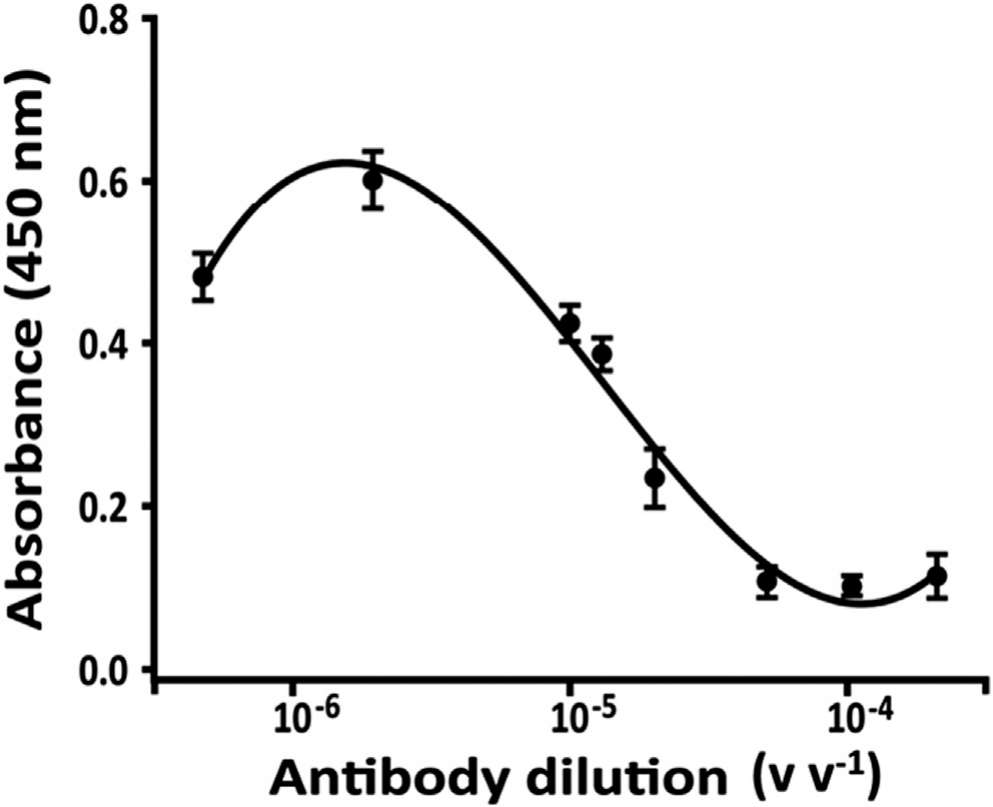
Binding curve for anti-STX PAb obtained with a fixed amount of STX-HRP (600 v v^−1^) in 20 mM phosphate buffer pH 7.4, after 2 h of incubation; flow rate, 0.15 ml min^−1^. The analysis was carried out using only bioreactor of the FI-IA system and the solution was collected.

The concentration of PAb to be used in the competition step is one of the very important parameters to be able to obtain an assay sensitive to low concentrations of STX. For the binding study (Figure 6), several dilutions of anti-STX PAb (1:500,000, 1:100,000, 1:75,000, 1:50,000, 1:20,000, 1:10,000, 1:5,000 v v^−1^, and no PAb, corresponding to the maximum of absorbance) and fixed amount of STX-HRP (1:600 v v^−1^) were incubated in different vials at room temperature. After 2 h (data not shown), the mixture was injected into the flow system with an increase of flow rate (0.15 ml min^−1^ flow rate instead 0.1 ml min^−1^, optimized for the only microchip) using a loop of 25 µL. A slight increase in the flow rate was necessary to create the right pressure for eluting the solution from the bioreactor and for transporting it inside to the microchip (due to the differences in the diameter of the tubes and channels, respectively).

After 3 min, a volume of 200 µL is eluted and collected in a vial in presence of 100 µL of TMB, the enzymatic substrate of HRP. After 1 min, the enzymatic reaction was blocked adding 100 µL of 1 mM H_2_SO_4_ causing the color turning from blue (TMox, 655 nm absorbance) to yellow (450 nm absorbance) and read spectrophotometrically.

In Figure 6, the absorbance is shown proportional to eluted STX-HRP from the bioreactor, not reacting with PAb (linked to G protein in the bioreactor) and inversely proportional to PAb. For the competition step, the selected dilution of PAb was equal to 1:75,000 v v^−1^, the 70% of the binding curve.

### Concentration of STX-HRP Using Bioreactor

To establish the dilution of STX-HRP to use for the competition step, several amounts of STX-HRP were added to fixed dilution of PAb (1:75,000 v v^−1^), extrapolated for the binding study (*Binding Curve and Competition Time Using Bioreactor*).

**FIGURE 7:**
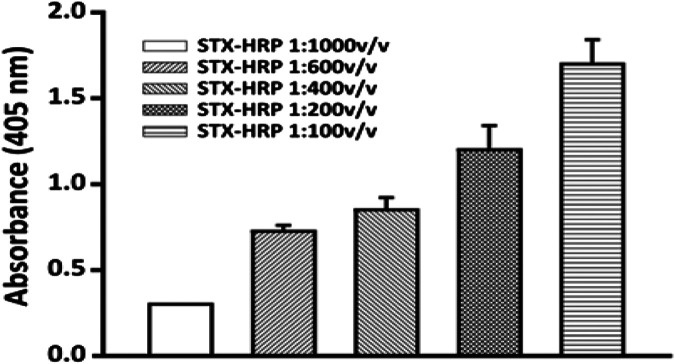
Selection of the STX-HRP dilution to use for the competition assay: 1:75,000 v v^−1^ dilutions of anti-STX PAb in 20 mM phosphate buffer pH 7.4, for 2 h at room temperature; flow rate, 0.15 ml min^−1^. The analysis was carried out using only the bioreactor of the FI-IA system.

The experiment was carried out using the same incubation times and procedures previously studied. Figure 7 reports the results obtained for several STX-HRP dilutions, where 1:600 v v^−1^ is selected (the absorbance respects the Lambert–Beer law with lower RSD% equal to 5%).

### Flow Optimization Using Bioreactor

The flow rate parameter has a significant influence on the retention of antigen-antibody complex by the G protein present in the bioreactor. Using the dilution selected in the previous study, 1:75,000 v v^−1^ of PAb and 1:600 v v^−1^ of STX-HRP, several flow rates are tested. Several flow rates were studied (Supplementary Figure S2), showing better results in terms of absorbance and reproducibility at 0.15 ml min^−1^.

### Calibration Curve Using the Bioreactor

**FIGURE 8:**
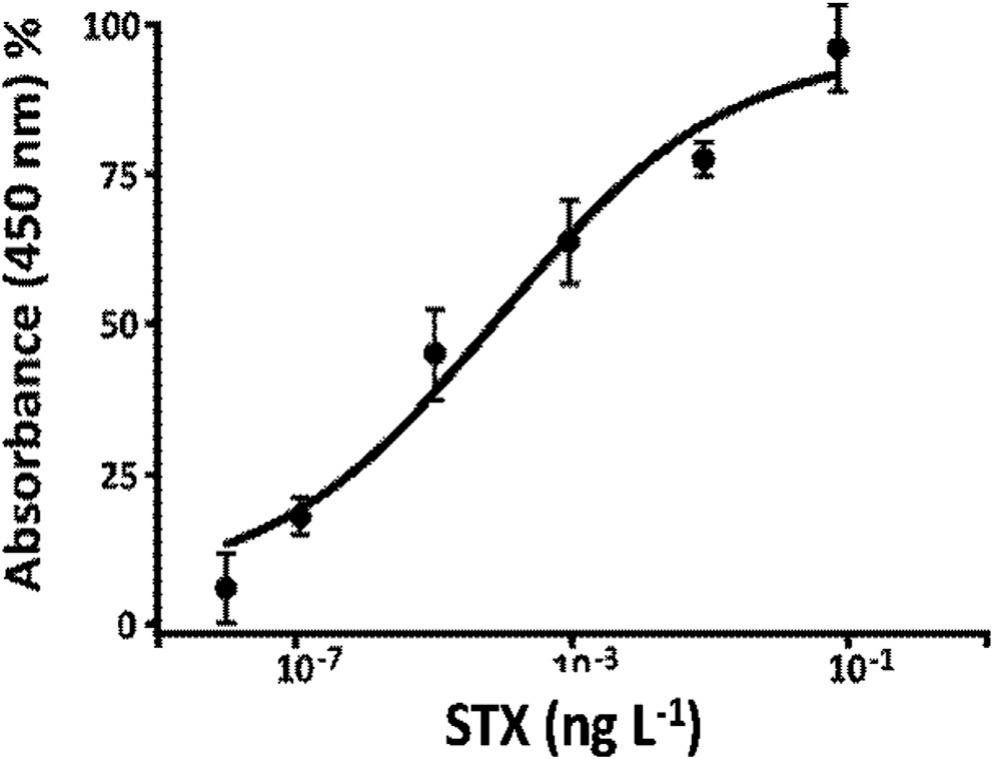
Calibration curve, using only bioreactor, for STX determination STX-HRP (1:600 v v^−1^) for the Anti-STX (1:75,000 v v^−1^) in 20 mM phosphate buffer pH 7.4, flow rate 0.15 ml min^−1^.

Calibration curve for STX determination in buffer (Figure 8) was obtained using the optimized parameters in the previous paragraph: 1:75,000 v v^−1^ of anti-PAB dilution, 1:600 v v^−1^ of STX-HRP, flow rate equal to 0.15 ml min^−1^, and competition time of 2 h at room temperature (data not shown). For this study, several concentrations of STX in buffer were prepared between 0 and 10^−1^ ng L^−1^. Every sample was prepared 15 min from each other in order to gain the best reproducibility due to the elution time of each solution and the washing time of the FI-IA system. A volume of 0.2 ml of samples was injected in the FI-IA system starting from the less concentrated solutions. After 3 min of elution time, the STX-HRP solution was collected in a vial with 100 µL of substrate TMB and left to react one more minute; the enzymatic reaction was blocked with H_2_SO_4_ lastly measured spectrophotometrically at 450 nm. Between measures, the system was washing in flow with 20 mM phosphate buffer NaH_2_PO_4_/Na_2_HPO_4_, pH 7.4, for 15 min, while the loop and the column were washed with approximately 0.4 ml of buffer every 5 min. The results indicate a “Hook Effect” (Supplementary Figure S3) for the higher concentrations of STX, a phenomenon due, probably, to the crowding near the antibody recognition sites and the difficulty of the toxin to be recognized for the formation of the immune complex. In fact, the signal response may decrease at extremely high concentrations as shown as a dip in the calibration curve range (Reverberi and Reverberi, 2007; Vashist and Luong, 2018). The results are demonstrated with a three-parameter logistic function trend, and the parameters are shown in Table 3 (calculated as reported in *FIA Peaks Analysis Applied to the Determination of STX*).

**TABLE 3:** Summary of results obtained with FI-IA system in diluted seawater samples.

	Bioreactor	Full system
LOD (ng ml^−1^)	5 × 10^–8^	1 × 10^–7^
WR (ng ml^−1^)	8 × 10^–7^–7 × 10^–5^	3.5 × 10^–7^–2 × 10^–5^
RSD%	12%	15%
Parameters	a = 0,9392; b = −0,2293; x_0_ = 2,523 × 10^–9^	b0 = 0.0357; b1 = 2.0635; b2 = −0.7858; r^2^ = 0.98

### Seawater Matrix Effect Using Bioreactor

**FIGURE 9:**
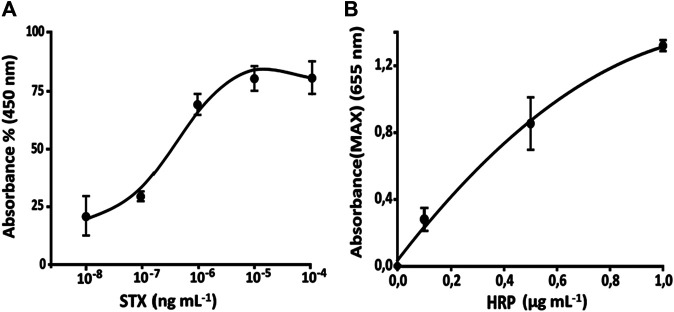
**(A)** Calibration curve for STX determination STX-HRP (1:600 v v^−1^) for the anti-STX (1:75,000 v v^−1)^ in diluted seawater (1:3 v v^−1^ with double distilled water) using bioreactor. (**B)** Calibration curve for STX obtained using microfluidic FI-IA system.

The system reliability was tested in a matrix matching method. The incubation protocol between STX and PAb in *Calibration Curve Using the Bioreactor* was performed in diluted seawater (1:3 v v^−1^ with double distilled water) and tested with different free STX concentrations. As a matter of fact, the matrix effect can influence the antigen-antibody interaction while performing analysis of STX concentration of 10^–7^, 10^–6^, 10^–5^, and 10^–4^ ng L^−1^ on seawater samples. Moreover, when concentrations values are higher than 10^–4^ ng L^−1^, a decrement of the signal (“Hook effect”) (Reverberi and Reverberi, 2007) may occur (Figure 9A) due to the crowding fo antigen vs. the antibodies immobilized on a small surface. The analytical parameters, extrapolated by the assay carried out in seawater, are reported in Table 3.

## Calibration Curve for the Full System (Bioreactor and Microfluidic Chip)

Regarding the results (Tables 3 **,** 4) of the last set (which correspond with the fully operational microfluidic system linked with the bioreactor), linearity is acceptable in relation to the used HRP concentrations (Figure 9B). Keeping in mind absorbance’s values for each HRP concentration, it is possible to observe (Figure 9) a lesser dispersion of the enzyme inside the bioreactor, thus causing a decrease of concentration in the mixing chamber between the first and last measure. This is probably due to lower enzyme mobility given by the column’s packing. This kind of system shows a LOD of 1 × 10^–7^, 3.5 × 10^–7^–2 × 10^–5^ ng mL^−1^ as working range, and an overall 15 RSD% (Table 3). Moreover, microfluidic chip’s structural features bind the working flow rate.

**TABLE 4:** Calibration curve parameters for the determination of STX using full system (bioreactor and microfluidic chip).

Concentration (µg ml^−1^)	**<**Absorbance**>**	RSD (%)
0	0.010 ± 0.003	30
0.1	0.28 ± 0.07	25
0.5	0.85 ± 0.16	19
1	1.319 ± 0.002	0.152

## Conclusion

This article outlines for the first time a novel FI-IA bioreactor connected with a microfluidic chip for the determination of STX, an important and harmful biotoxin present in seawater. The proposed system gives the possibility of analyzing in several continuous samples, reading in an automated way the results. The resin bead–based bioreactor sample capacity allows analysis of up to 38 sequential samples to be performed before it needs to be changed. The preliminary study, presented here, illustrates its potential to being used as a field-deployable portable analytical device able to work *in situ* and with continuous water samples without any pretreatment for monitoring the health and safety status of marine environments tool to warn and protect against the consumption of contaminated marine produce.

## Data Availability

The original contributions presented in the study are included in the article/Supplementary Material; further inquiries can be directed to the corresponding authors.
